# Causal pathway from telomere length to occurrence and 28-day mortality of sepsis: an observational and mendelian randomization study

**DOI:** 10.18632/aging.204937

**Published:** 2023-08-04

**Authors:** Tao Jiang, Xuan Mo, Ruonan Zhan, Yi Zhang

**Affiliations:** 1 Department of Anesthesiology, Hubei Key Laboratory of Geriatric Anesthesia and Perioperative Brain Health, and Wuhan Clinical Research Center for Geriatric Anesthesia, Tongji Hospital, Tongji Medical College, Huazhong University of Science and Technology, Wuhan, Hubei, People’s Republic of China; 2 Department of Anesthesiology, The First Affiliated Hospital of Anhui Medical University Gaoxin District, Hefei, Anhui, People’s Republic of China

**Keywords:** telomere, sepsis, 28-day mortality, mendelian randomization, SNPs

## Abstract

Background: Telomeres are considered to be a physiological marker of aging. Elucidating relationship between telomere length and sepsis is an essential step towards understanding the biological processes involved in sepsis and its salvation. Mendelian randomization studies based on SNPs have given us new insights into genetic susceptibility to disease.

Objectives: To explore the causal pathway from telomere length to occurrence and 28-day mortality of sepsis.

Methods: Leveraging genetic information resource of UK Biobank, we captured three groups of large-scale GWAS data: leukocyte telomere length (LTL), sepsis and all-cause death of 28-day. Study design consisted of three parts: forward analysis, reverse analysis and one-way analysis. Genetic instrumental variables were selected for different analyses under the premise that three MR core assumptions were satisfied. Causality was determined by means of IVW.

Results: In forward analysis, we did not observe a significant causal pathway from sepsis to LTL under IVW model: β (SE) was −0.0051 (0.0075) with a *p*-value of 0.499. In reverse analysis, based on the IVW model, the OR (95% CI) was 0.89 (0.80–0.99) and the *p*-values was 0.043; based on the results of leave out method and single SNP analysis, we obtained seven key SNPs. There were results of IVW model in the one-way analysis: β (SE) was −0.0287(0.1261).

Conclusions: Short LTL increases susceptibility to sepsis, but sepsis does not shorten telomere length. LTL does not affect sepsis 28-day all-cause mortality and does not serve as a causal intermediate in gene regulation during the progression of sepsis to 28-day death.

## INTRODUCTION

It has been reported that 27% of ICU admissions in the UK are for patients with sepsis [1]; in the United States, 18.6 out of 10,000 people are diagnosed with sepsis each year [2]; globally, the value is 43.7 and in-hospital mortality rate is 17%, with a conservative estimate of 5.3 million deaths per year due to this critical condition [3]. As one of the leading causes of high mortality and increased health care costs in modern intensive care units, sepsis is a global challenge [4, 5]. Notably, elderly population is at high risk for sepsis and patients with a clinical diagnosis of sepsis have a very high mortality rate within 28 days. Under the condition of severe infection or sepsis, the immune response is often impaired and the inflammatory process is out of control [6]; oxygen radicals are at a high level, thus affecting cell physiological functions, even genetic material [7].

Telomeres, as important structures of chromosomes [8], are of great significance in DNA protection and chromosome stability, although no role has been found for DNA repair. What’s more, telomeres are considered to be a physiological marker of aging [9]. In addition to aging and other genetic factors, environmental agents, such as radiation, ozone exposure, and lifestyle, can cause changes in telomere function. Dysfunction of telomere biology is directly reflected in the variation of length. These alterations are thought to be associated with risks for a variety of age-related diseases [10], including cardiovascular disease [11, 12], cancer [13], and psychiatric disorders [14]. Most importantly, the maintenance of telomere biological function has been used in treatments of diseases such as chronic kidney disease [15].

Elucidating the relationship between telomere length and sepsis is an essential step towards understanding the biological processes involved in sepsis and its salvation. Two prospective observational studies showed that shorter leukocyte telomere length (LTL) was associated with a higher risk of infection [16] and a lower 90-day survival rate in sepsis patients [17], respectively. However, there is still a major gap in understanding. Due to residual confounding variables (such as concomitant underlying diseases) or reverse causality, it is unclear whether telomere length shortening is an incidental phenomenon of sepsis or telomere length is causally related to sepsis and septic death.

Traditionally, the determination of causality has relied only on high-quality randomized controlled studies. Now, mendelian randomization (MR) studies based on single nucleotide polymorphisms (SNPs) can provide stronger evidences to verify causal associations than observational studies [18, 19]. The emergence probability of a particular genetic information regarding the exposure factor (assigned by random combination of previous generation alleles) is correlated with the outcome event, then this principle offers the possibility to uncover causal effects between things [20].

Genome-wide association studies (GWAS) have given us new insights into genetic susceptibility to disease. In this research, a two-sample bidirectional MR design is adopted to make full use of the genetic data collected by GWAS. And the aim is to explore the causal pathway from telomere length to occurrence and 28-day mobility of sepsis.

## MATERIALS AND METHODS

### Datasets

GWAS data of leucocyte telomere length were all obtained from a European population of 472,174 participants (UK Biobank, https://figshare.com/s/caa99dc0f76d62990195), and data information can be found in Supplementary Table 1. The ratio of telomere repeat number (T) to single-copy gene (S) (T/S ratio) was obtained by qPCR, and LTL (Field 22190) was adjusted accordingly (quasi-normal distribution transformation and Z-normalization) to facilitate comparison with other datasets. Full details of the technical adjustments can be found at https://doi.org/10.1101/2021.03.18.21253457.

GWAS data for both sepsis and septic 28-day all-cause death were obtained from the same European population, including 486,484 participants. As shown in Supplementary Table 1, 11,643 patients with sepsis were clinically diagnosed and 2,243,539 SNPs were obtained (UK Biobank, https://gwas.mrcieu.ac.uk/datasets/ieu-b-4980/); the number of 28-day death patients was 1896, with those who survived being used as controls, then 1,243,487 SNPs were obtained (UK Biobank, https://gwas.mrcieu.ac.uk/datasets/ieu-b-5064/). Data were corrected to avoid potential bias from population stratification, such as age and gender, and inaccuracies caused by technical differences.

### Genetic instrumental variables

**Figure 1:**
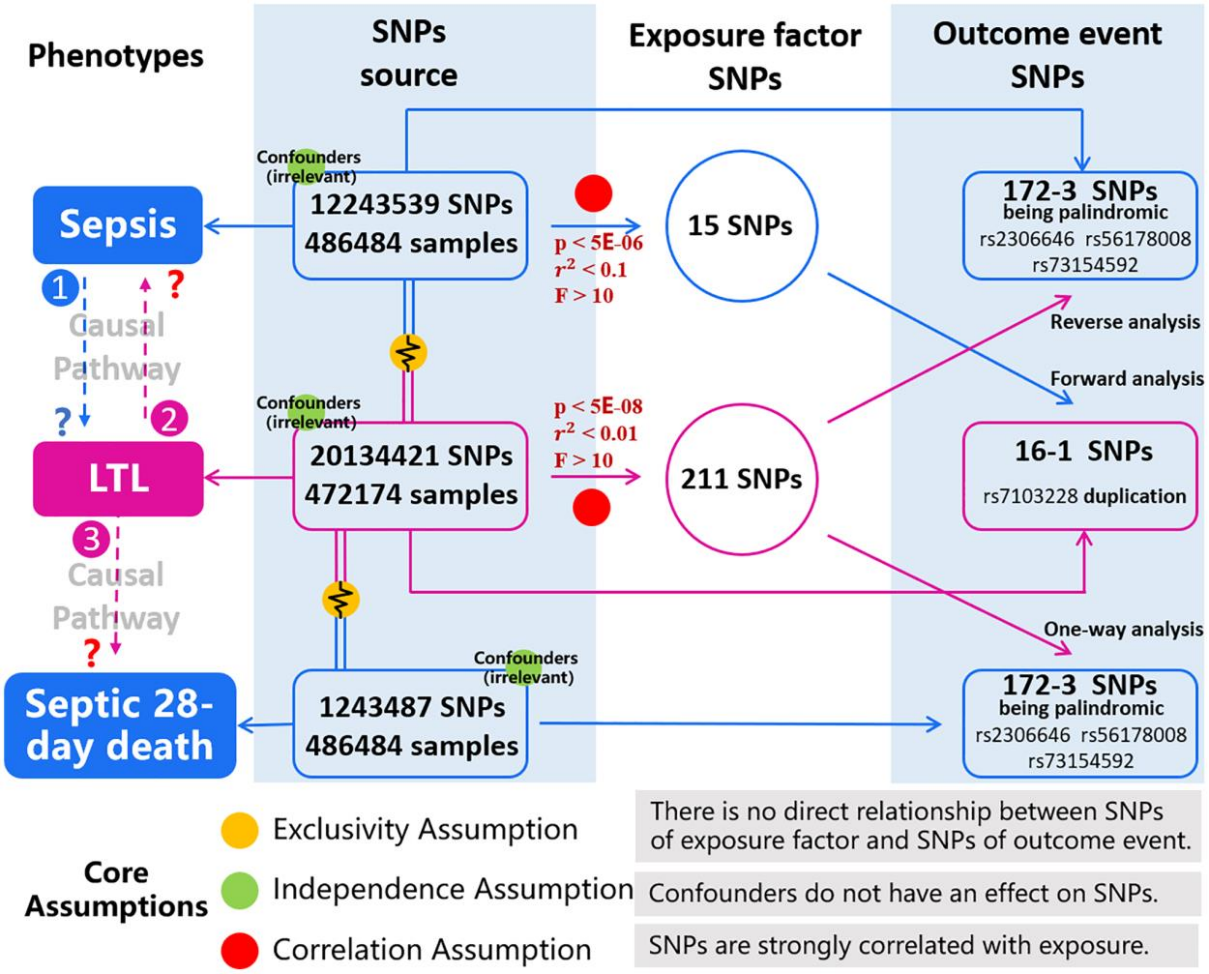
**Description of the study design in this MR study.** Study design consists of three parts: forward analysis (sepsis to LTL), reverse analysis (LTL to sepsis) and one-way analysis (LTL to septic 28-day death). In the population cohort of exposure factor we obtain SNPs (consistent with Exclusivity and Independence assumptions), and according to the Correlation assumption, we obtain SNPs of exposure factors. In another cohort of outcome event, we obtain SNPs, and we can extract outcome-related SNPs by matching with SNPs of exposure factor. Correlation between SNPs in both groups is analyzed, then correctness of causal pathway is judged. Three types of MR analysis correspond to the judgment of three causal pathways: Causal Pathway 1, people with sepsis have a tendency to shortening leukocyte telomeres; Causal Pathway 2, people with shorter LTL increase the susceptibility to sepsis; Causal Pathway 3, people with shorter LTL are more likely to die of sepsis within 28 days. The blue represents the phenotypes and SNPs involving in sepsis and 28-day death. The pink represents phenotypes and SNPs of LTL. The red illustrates threshold conditions of genetic instrumental variables. Yellow, green, and red balls represent the core assumptions of MR analysis. Abbreviations: LTL: leukocyte telomere length; SNPs: single nucleotide polymorphisms.

To illustrate the workflow of the study, the study design is shown in Figure 1, which consists of three parts: forward analysis, reverse analysis and one-way analysis. In the forward analysis and reverse analysis, sepsis and LTL were exposure factors respectively; in the one-way analysis, LTL was the exposure group and 28-day death was the outcome group of the study. Three types of MR analysis correspond to the judgment of three causal pathways: Causal Pathway 1, people with sepsis have a tendency to shortening leukocyte telomeres; Causal Pathway 2, people with shorter LTL increase the susceptibility to sepsis; Causal Pathway 3, people with shorter LTL are more likely to die of sepsis within 28 days. Researchers needed to select the corresponding genetic instrumental variables that met the conditions in each of the two epistatic traits (sepsis and LTL).

MR must rely on three core assumptions: (1) Exclusivity Assumption; (2) Independence Assumption; and (3) Correlation Assumption. As shown in Figure 1, there is no direct relationship between SNPs corresponding to the exposure factor and the outcome event, as well as confounders do not have an effect on SNPs. Therefore, the first two assumptions (Exclusivity and Independence) have been satisfied in this study. In terms of Correlation Assumption, it requires that SNPs are strongly correlated with exposure. We calculated r^2^, which indicates the proportion of variance in exposure variables explained by genetic variation; we estimated the F statistic to assess the strength of the association between SNPs and exposure factors [21, 22]. In the case of sepsis as an exposure element, before SNPs can be considered as potential tools, the following threshold conditions needed to be met: *p* value < 5E-06, distance > 1Mb, r^2^ < 0.1 (linkage disequilibrium clustering), and F > 10. When LTL was used as an exposure factor, the characteristic SNPs were significantly different and numerous, then we set more stringent threshold conditions: *p* value < 5E-08, distance > 1Mb, r^2^ < 0.01, and F > 10.

Quasi genetic tools (potential tools) became genetic instrumental variables, which needed to go through the following process: harmonization between SNPs of exposure and those of result.

And there may be loss of characteristic SNPs in this process: (1) Not all SNPs of exposure can be paired with SNPs of result; (2) There were duplicate SNPs, but common sites had different bases, that is, there were multiple effect sites; (3) Being palindromic with intermediate allele frequencies.

### Statistical analysis

All analyses were performed using the R (version 4.2.0, http://www.rproject.org/) runtime environment, and “TwoSampleMR” package and “MendelianRandomization” package were obtained for use. Regardless of forward analysis, reverse analysis or one-way analysis, causal effect estimation mainly relied on the random-effects inverse-variance weighted estimation method (IVW). In fact, there may be invalid SNPs that bias the judgment of causality. For the above one reason, we used three methods, MR Egger, Weighted median and Weighted mode, for sensitivity analysis. In the case of horizontal pleiotropy, these three models would give more reliable results, although at the cost of reduced statistical capacity. And finally, causal estimates, *p* value, β value and standard error (SE) were obtained for both categorical and continuous variable outcomes. *P* < 0.05 indicated that there was a causal effect relationship. Moreover, for binary variables, odds ratio (OR) and 95% CI were used to estimate the degree of causality.

### Exploration of pleiotropy and heterogeneity

All the following statistical analyses were performed using R (version 4.2.0, https://www.rproject.org/) and related software packages (“TwoSampleMR” and “MR-PRESSO”). According to the effect value and standard error of different SNPs in the outcome events (LTL, sepsis and 28-day death), funnel plots were drawn to explore the overall heterogeneity and SNPs that could introduce bias. For one thing, the strategy of leave-one-out method checked the potential impact of each outlier SNP on the overall cause and effect estimation, then it helped us to find key SNPs; by means of single SNP analysis, we can determine whether single SNP influences the main causality of the MR study. In addition, MR-Egger intercept test, modified Cochran Q statistic and MR-pleiotropy residual sum and outlier (MR-PRESSO) [23] were also used to detect the robustness of the significance results. Of note, when MR-PRESSO analysis was performed for outcome events (LTL, sepsis and 28-day death), Nb Distribution was required to be set to 1000, 4000 and 2000, respectively, to achieve an effective threshold of *p* < 0.05.

## RESULTS

### Causal effect of sepsis on LTL (causal pathway 1)

In forward analysis, the potential genetic tools that met the threshold conditions were 15 SNPs, which were confirmed to be related to sepsis. These quasi-genetic tools were used to reconcile LTL-related independent loci, then 16 SNPs were screened. After excluding a duplicate SNP (rs7103228), 15 independent SNPs were considered as powerful genetic tools in the study. See Supplementary Table 2 for details of specific summary data.

**Figure 2:**
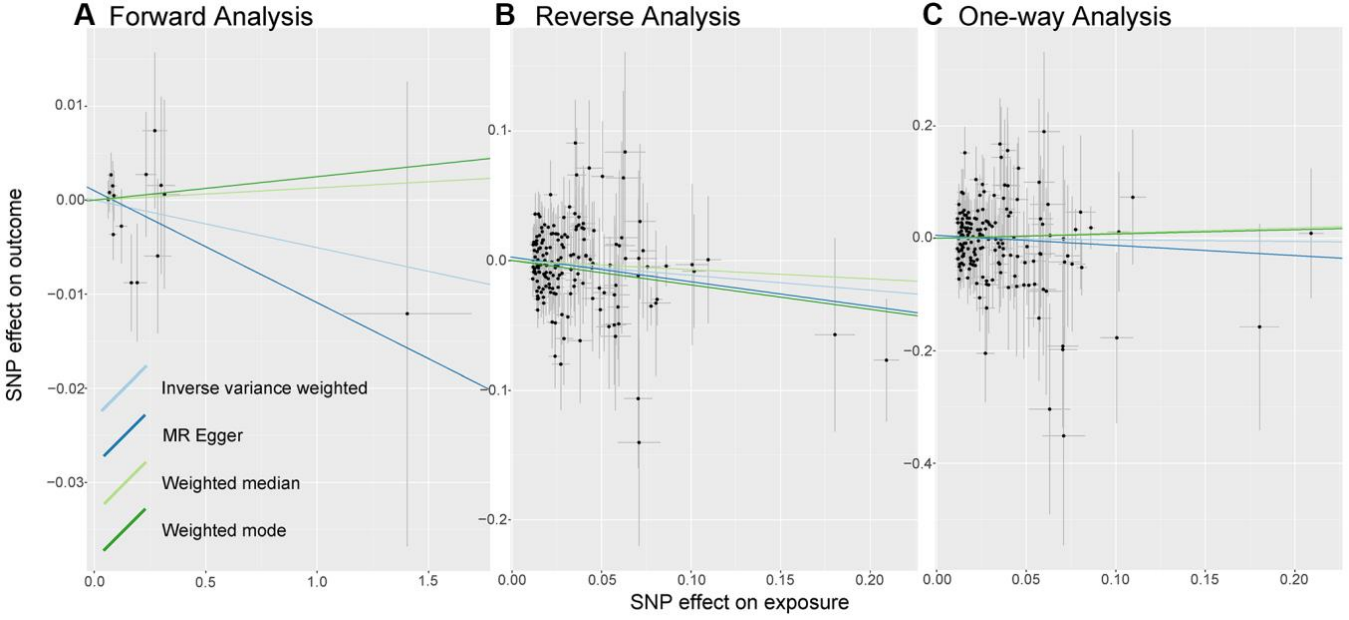
**Analysis of MR.** There are scatter plots of the association between exposure SNP effect and outcome one. Analysis is conducted using the conventional inverse-variance-weighted MR method and complementary methods, including MR-Egger, weighted median, weighted mode approaches. Causal judgment depends on slope after each linear fitting. (**A**) Used to determine the causal effect of sepsis on LTL. (**B**) Utilized to determine the causal effect of LTL on sepsis. (**C**) Designed to judge the causal effect of LTL on 28-day mortality. Abbreviations: LTL: Leukocyte Telomere Length; MR: Mendelian randomization; SNP: single-nucleotide polymorphism.

As shown in Figure 2A, we do not observe a significant causal relationship between sepsis and LTL under the IVW model: β (SE) is −0.0051 (0.0075) with a *p*-value of 0.499. What’s more, the results of the three models, MR Egger, Weighted median and weighted mode, are consistent with those of IVW (Table 1). It is worth noting that 95% CI became closer near zero, which increased the credibility of non-causality discovery.

**Table 1:** Results of mendelian randomization analysis.

**Exposure**	**Outcome**	**Methods**	**OR (95% CI)**	**Β (SE)**	***P* value**
Forward Analysis					
Sepsis	LTL	IVW	–	−0.0051 (0.0075)	0.499
		MR Egger	–	−0.0119 (0.0127)	0.366
		Weighted median	–	0.0013 (0.0104)	0.901
		Weighted mode	–	0.0025 (0.0149)	0.870
Reverse Analysis					
LTL	Sepsis	IVW	0.89 (0.80–0.99)	−0.1143 (0.0566)	0.043
		MR Egger	0.83 (0.68–1.01)	−0.1894 (0.1001)	0.060
		Weighted median	0.93 (0.78–1.11)	−0.0706 (0.0881)	0.423
		Weighted mode	0.83 (0.69–1.00)	−0.1885 (0.0956)	0.050
One-way Analysis					
LTL	Septic 28-day death	IVW	0.97 (0.76–1.24)	−0.0287 (0.1261)	0.820
		MR Egger	0.84 (0.54–1.29)	−0.1801 (0.2231)	0.421
		Weighted median	1.10 (0.73–1.65)	0.0912 (0.2088)	0.662
		Weighted mode	1.08 (0.67–1.73)	0.0760 (0.2415)	0.753

Abbreviations: CI: confidence interval; IVW: inverse variance weighted; LTL: leukocyte telomere length; MR: Mendelian randomization; OR: odds ratio; SE: standard error.

### Causal effect of LTL on sepsis (causal pathway 2)

We selected genetic instrumental variables for reverse analysis from 211 independent loci associated with LTL. 39 of those SNPs were missing in the GWAS of sepsis. Also, we removed being palindromic with intermediate allele frequencies: rs2306646, rs56178008, and rs73154592. Therefore, we finally included 169 polymorphic variants as genetic tools in the reverse MR analysis (Supplementary Table 3).

In the causal inference analysis, we find that shorter LTL would increase susceptibility to sepsis (Figure 2B). As shown in Table 1, based on the IVW model, the OR (95% CI) is 0.89 (0.80–0.99) and the *p*-value is 0.043. Additionally, although in the MR-Egger, Weighted median model and Weighted Mode model the association is not statistically significant (all *p*-value are more than 0.05), the values of β (SE) are −0.1894 (0.1001), −0.0706 (0.0881), and −0.1885 (0.0956). What’s more, the directions of other models remain consistent with IVW. The results we observe support the idea that there is a causal relationship between LTL and sepsis.

### Causal effect of LTL on 28-day mortality (causal pathway 3)

Genetic instrumental variables included in reverse analysis and one-way analysis were identical. 184 SNPs were utilized as instrumental variables for telomere length to participate in one-way MR analysis. See Supplementary Table 3 for details.

As shown in Figure 2C, there is no evidence of a causal trend between genetically determined LTL and 28-day death. As shown in Table 1, after linear data fitting by IVW, MR-Egger, Weighted Median and Weighted Mode, β (SE) is −0.0287 (0.1261), −0.1801 (0.2231), 0.0912 (0.2088), and 0.0760 (0.2415), respectively. 95% confidence intervals for OR are wide, with *p*-values greater than 0.05, then associations are not shown to be significant in different models.

### Exploration of pleiotropy and heterogeneity

**Figure 3:**
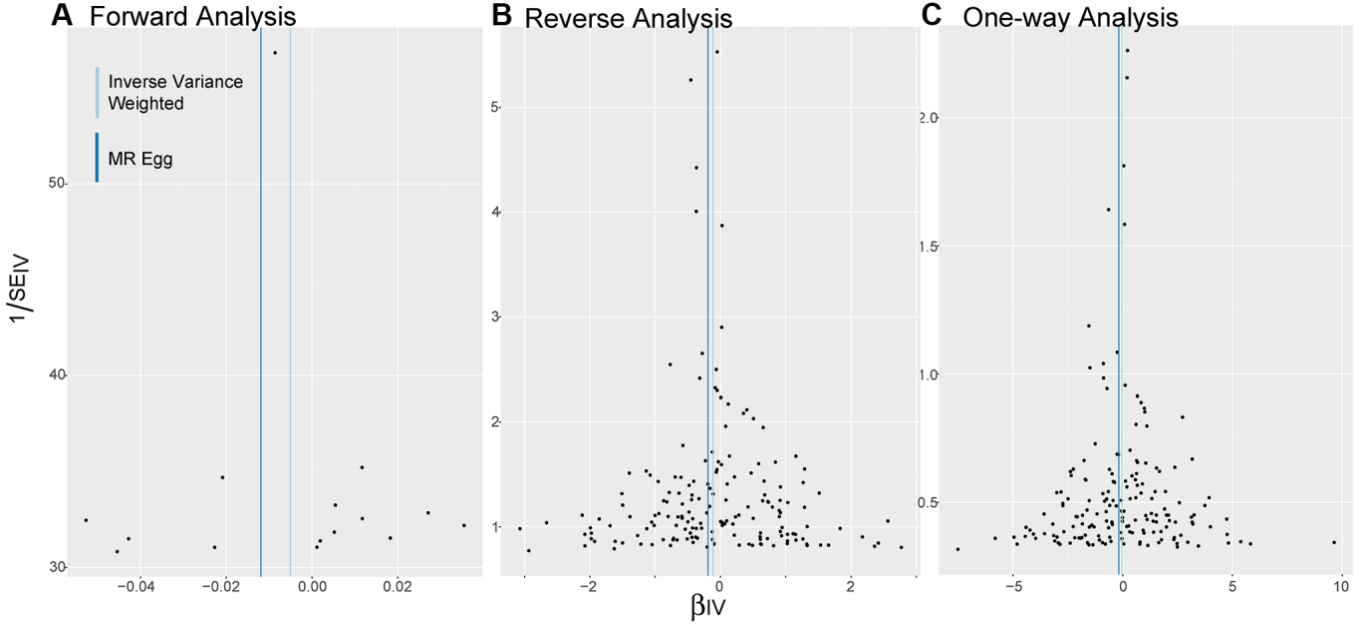
**Funnel plots.** (**A**–**C**) Are applied to detect whether the observed associations of different analyses were along with obvious heterogeneity. Distribution of SNPs as genetic tools is symmetrical on both sides of the IVW line and MR Egger line. Abbreviations: IV: instrumental variables; IVW: inverse variance weighted; SE: standard error.

As shown in Figure 3, the distribution of SNPs as genetic tools is symmetrical on both sides of the IVW line and MR Egger line, no matter in bidirectional MR analysis or one-way analysis.

**Figure 4:**
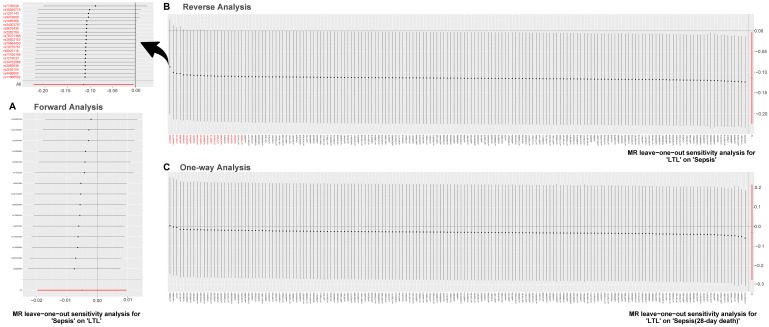
**MR leave-one-out sensitivity analysis.** Leave-one-out analysis is used to evaluate whether any SNP is driving the causal effect. (**A**) Represents the results of MR leave−one−out sensitivity analysis for “Sepsis” on “LTL”. (**B**) Reflects the findings of MR leave−one−out sensitivity analysis for “LTL” on “Sepsis”, which change dramatically when SNPs marked in red are removed. (**C**) Contains the results of MR leave−one−out sensitivity analysis for “LTL” on “Sepsis (28-day death)”. Abbreviations: LTL: leukocyte telomere length; MR: Mendelian randomization; SNPs: single-nucleotide polymorphisms.

**Figure 5:**
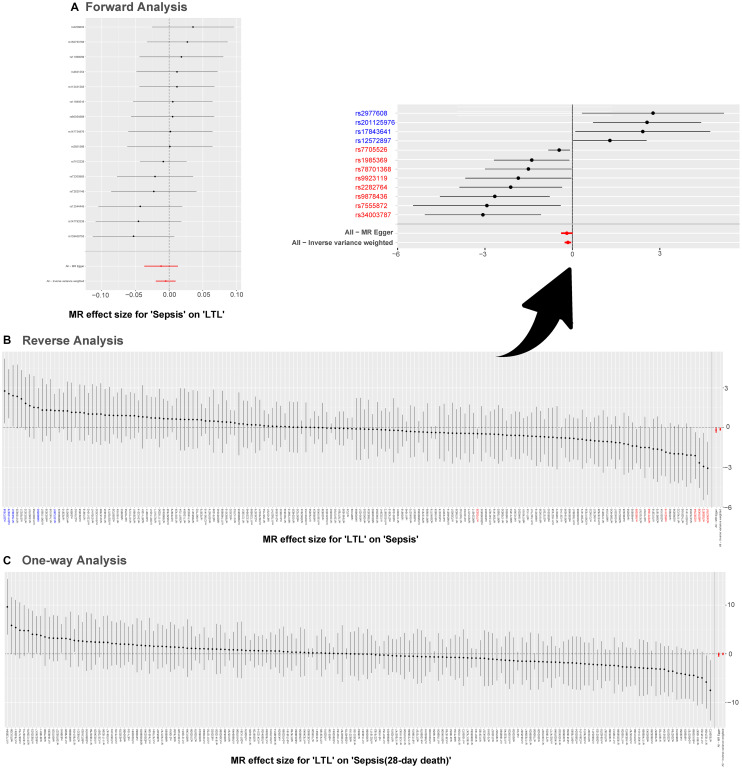
**Single SNP analysis of MR.** (**A**–**C**) are used to show the MR estimate and 95% CI values for each SNP, also show the IVW and MR-Egger MR results at the bottom. In (**B**), SNP marked in red supports the result that short LTL increases the susceptibility to sepsis; the blue marked SNP rejects the MR result. Abbreviations: LTL: leukocyte telomere length; MR: Mendelian randomization; SNPs: single-nucleotide polymorphisms.

Results of the leave-one-out method can be seen in Figure 4. In Figure 4A, 4C, no single SNP drives these results, and correlations do not change significantly, which confirms the stability of results in both forward analysis and one-way analysis. Surprisingly, in Figure 4B, there exist 20 SNPs: rs1291143, rs35640778, rs7705526, rs9419958, rs1985369, rs34003787, rs9878436, rs2282764, rs78701368, rs28502153, rs75664430, rs79755767, rs9923119, rs77426195, rs7218033, rs34762068, rs2069536, s2555104, rs4498805 and rs11866592. Elimination of these have a dramatic impact on the overall result: correlation shifts significantly. Results of the Single SNP analysis of MR can be seen in Figure 5. The vast majority of SNPs estimate crossed a value of 0, indicating that the results were not significant. However, it is noteworthy that there are 12 SNPs evaluated in the reverse analysis with significantly different results. The marked red SNPs (rs34003787, rs7555872, rs9878436, rs2282764, rs9923119, rs78701368, rs1985369 and rs7705526) support the result of MR analysis; the marked blue those (rs12572897, rs17843641, rs201125976 and rs2977608) reject it. Based on the results of leave out method and single SNP analysis, we obtain seven SNPs (overlap): rs7705526, rs1985369, rs34003787, rs9878436, rs2282764, rs78701368, rs9923119. The genomic loci variants mapped by these SNPs may have an unusual role in the mechanism of increased susceptibility to sepsis by short LTL.

MR Egger intercept test is used to evaluate directional pleiotropy. The confidence interval of the intercept contains 0, and the *P* values are greater than 0.05 (Table 2), indicating that there is no potential incidence of directional pleiotropy. On the other hand, MR-PRESSO judgment of horizontal pleiotropy is equal to and diverged from the former: *p* values of Global Test for forward analysis and one-way analysis are 0.828 and 0.223 respectively; however, in reverse analysis, *P* value is 0.027, which suggests that the analysis may be influenced by serious outliers.

**Table 2:** Pleiotropy and heterogeneity analysis.

**Design**	**MR-Egger regression**		**MR PRESSO_global test**	**Heterogeneity analysis**		
	**Intercept (SE)**	***P* val**	***P* val**	**Method**	***Q***	***Q_P* val**
Forward Analysis	0.0010 (0.0015)	0.516	0.828	IVW	10.32	0.738
				MR-Egger	9.878	0.704
Reverse Analysis	0.0026 (0.0028)	0.365	0.027	IVW	202.6	0.035
				MR-Egger	201.6	0.035
One-way Analysis	0.0052 (0.0063)	0.412	0.223	IVW	168.5	0.474
				MR-Egger	167.8	0.467

Abbreviations: IVW: inverse variance weighted; MR: Mendelian randomization; MR PRESSO: Mendelian randomization pleiotropy residual sum and outlier; *P* val: *p* value; SE: standard error.

Test of heterogeneity often relies on modified Cochran Q statistic. As shown in Table 2, there is no significant heterogeneity between forward analysis and one-way analysis (*P* > 0.05 for IVW model and *P* > 0.05 for MR-Egger model). One issue is recognized in Reverse Analysis: overall heterogeneity is large. However, the heterogeneity is acceptable in this study using IVW with random effects as the primary outcome.

## DISCUSSION

Leveraging the powerful genetic information resource of UK Biobank [24, 25], we captured three groups of large-scale GWAS data (LTL, sepsis and all-cause death of 28-day); both bidirectional two-sample MR analysis was drawn upon to assess the causal association between telomere length and sepsis, and unidirectional two-sample MR analysis was employed to determine the notion that short telomere length promoted high 28-day mortality in sepsis. Through SNPs as a proxy of LTL, MR analysis supported the evidence from traditional analysis that telomere length shortening led to increased susceptibility to sepsis. However, no evidence was found to favor the following two ideas: sepsis can make LTL shorter; and LTL had a significant genetic correlation with septic 28-day mortality. On the basis of the above findings we proposed an interpretation that telomere length factors did not work as cause-effect intermediates of gene regulation during the progression of sepsis to death within 28 days.

### Forward analysis

In a previous animal trial, researchers found that sepsis induced telomere shortening in various tissues and suggested that this phenomenon could be a potential mechanism for delaying pathophysiological events in sepsis survivors [26]. Interestingly, in a prospective observational study with a small sample, sepsis patients showed changes in telomere length (shortening and lengthening) and the trends in shortening were not statistically different [27]. There are numerous potential factors affecting TL, such as smoking, alcohol consumption, ozone exposure, cell type, and telomere measurement techniques. The present study used MR analysis, exempting confounding factors and reverse causality, to further strengthen the causal link of the above view: the occurrence of sepsis cannot lead to telomere length shortening.

### Reverse analysis

In a prospective observational study of 75,309 individuals from the general population, shorter LTL was associated with a higher risk of infection, but after subgroup analysis, there was no correlation between telomere length and the risk of sepsis [16]. More complicated, our reverse analysis found that it was inconsistent with this traditional observational study: the former tended to report that shortened telomere length would increase the susceptibility to sepsis (causally); in the latter study (observational one), according to our forward analysis findings, we can assume that the reverse causal link (sepsis to LTL) does not exist, so we are more willing to attribute the contrary conclusion to the interference of confounders.

Clinically, shorter LTL increases the propensity from infection to sepsis. On the one hand, telomeres, as biomarkers of aging and frailty, are involved in numerous age-related diseases [28]; on the other hand, telomeres may play an important role in immune inflammatory diseases [29]. Telomere length determines the number of replications and lifespan of the cell. Immune cell function gradually decreases with age, and this immune aging [30] is closely related to telomere length. Susceptibility to infection arises from immune aging [31]. In a prospective cohort study, a large proportion of elderly patients with severe infections had higher inflammatory protein abundance and lower lymphocyte counts [32]; in a retrospective cohort study on the analysis of risk factors for postoperative sepsis in elderly patients, an abnormal neutrophil-to-lymphocyte ratio was clearly identified as a risk factor [33]. The above findings corroborate the presence of immune senescence. As a result, severe infections are more inclined to sepsis.

Genetically, age-related telomere attrition can lead to aberrant gene expression in sub-telomere regions [34], and polymorphisms in such genes may trigger the development of sepsis susceptibility [35]. This research was conducted to verify the causal interaction between LTL and sepsis with the help of single nucleotide polymorphisms. rs9878436 (chr3:138525558), rs2282764 (chr4:2253336), rs7705526 (chr5:1285859), rs1985369 (chr7:159326530), rs34003787 (chr16:73037482), rs9923119 (chr16:90087407) and rs78701368 (chr20:63834076) were relatively heavily weighted in the integrated results and had a dramatic bearing on the final outcome. In previous studies, rs7705526 revealed immune-related loci for risk of systemic lupus erythematosus [36], monoclonal hematopoiesis [37], and LTL variants with risk of hypertension and coronary heart disease [38]. The upstream and downstream genes of single nucleotide polymorphisms and gene polymorphisms are involved in the occurrence of diseases and the regulation of cellular functions. Now, it is plausible to suggest that genes up or down seven previously mentioned loci may be instrumental in the mechanism by which short LTL increases vulnerability to sepsis.

### One-way analysis

Historically, the relationship between telomere length and mortality of sepsis was fraught with uncertainty. Supporting evidence existed. In a retrospective cohort of US adults, an increase of telomere length was in association with a decrease of all-cause mortality but did not reveal an association between LTL and sepsis-specific mortality [39]; in a longitudinal follow-up study of 72,432 UK participants, shorter LTL correlated with a smaller increased risk of overall mortality [40]. Negative views were not absent. Multiple variables accurately predicted 30-day mortality in the cohort of patients with severe infections, but the age variable was excluded [41]. In the Toledo Study for Healthy Aging and ENRICA cohorts with long follow-up, TL did not significantly alter the risk of death and predict mortality in older adults [42].

Presently, in our analysis, telomere length is represented by 169 genetic variants, avoiding the bias present in the aforementioned studies. Consequently, MR approach can yield a more reliable estimate for this view than the traditional observational one: there is no causal path between genetically predicted LTL and septic 28-day mortality.

### Clinical relevance of findings

MR analysis methods facilitate the understanding and treatment of sepsis by healthcare professionals [43]. Although LTL does not alter sepsis 28-day mortality, long LTL can impede the progression of infection to sepsis, which can provide potential clinical applications. First, telomere length can be used as a recognition factor. For infected patients, clinical staff can determine LTL to identify high-risk groups of sepsis and establish an early warning system for sepsis, which can greatly improve the prognosis of patients and reduce the cost of sepsis treatment [44, 45]. Secondly, telomere length can be used as a target for intervention when appropriate. In the face of infection in critically ill patients, in addition to antibiotics, telomere shelter measures, such as Omega-3 fatty acids [46], selenium and coenzyme Q10 [47, 48], may bring unexpected therapeutic effects.

### Study limitations

When we interpret the findings of this study, limitations must be taken into account. First of all, population type and group stratification should be emphasized in MR analysis. Data were obtained from European populations, so the extrapolation of the findings should be treated with caution. With regard to group stratification, SNPs data of LTL did not consider the impact of gender, but in other aspects, such as chips and detection technology, researchers have made corrections; SNPs related to sepsis and 28-day mortality were adjusted for age, sex, and testing methods before enrollment. Second, the relationship between telomeres and sepsis is complex. However, MR can only be fitted linearly to explore causal effects based on the parameters entered in the model. This approach would allow us to ignore nonlinear relationships. Finally, TL in this study were from leukocytes and the length of telomeres in other tissue cells were not considered. Considering the presence of cell storms in the blood of patients with sepsis, LTL may be the best choice.

## CONCLUSION

Conclusively, short LTL increases susceptibility to sepsis, but sepsis does not shorten telomere length. Telomere length does not affect sepsis 28-day all-cause mortality and does not serve as a causal intermediate in gene regulation during the progression of sepsis to death within 28 days.

## Supplementary Materials

Supplementary Tables 1 and 2

Supplementary Table 3
