# Supplementary material for: Causal pathway from telomere length to occurrence and 28-day mortality of sepsis: an observational and mendelian randomization study
Source: Aging (Albany NY). 2023 Aug 4;15(15):7727–40. doi: 10.18632/aging.204937 (PMC10457059; doi:10.18632/aging.204937)
Supplement: Supplementary Tables 1 and 2 [file aging-15-204937-s001.pdf]

SUPPLEMENTARY TABLES

Supplementary Table 1. Information for data source.

Factors	Study name	Population	No. cases	No. controls	No. of SNPs	Sample size	Source	Note
Sepsis	UK Biobank	European	11643	474841	12243539	486484	https://gwas.mrcieu.ac.uk/datasets/ieu-b-4980/	adjusted for age, sex, chip, and the first 10 PCAs.
Sepsis (28-day death)	UK Biobank	European	1896	484588	1243487	486484	https://gwas.mrcieu.ac.uk/datasets/ieu-b-5064/	adjusted for age, sex, chip, and the first 10 PCAs.
Leucocyte Telomere length	UK Biobank	European	-	472174	20134421	472174	https://figshare.com/s/caa99dc0f76d62990195	NA

Supplementary Table 2. Genetic instrumental variables of forward analysis.

SNP	Sepsis_exposure						LTL_outcome					Note
	Effect Allele	Other Allele	Beta	Se	*p*_value	F	Effect Allele	Other Allele	Beta	Se	*p*_value	
rs11068069	C	T	0.082983	0.01791	3.60E-06	1082.41	C	T	0.001518	0.002632	0.56	Not Reported in ClinVar
rs112431283	T	A	−0.23295	0.046156	4.49E-07	1017.83	T	A	0.002725	0.006614	0.68	ADRA1B: Intron Variant
rs11980516	G	T	0.088518	0.018058	9.50E-07	1268.21	G	T	0.000485	0.002662	0.86	Not Reported in ClinVar
rs12544445	C	T	−0.08549	0.01829	2.95E-06	1009.11	C	T	0.003658	0.002714	0.18	LOC101927066: Intron Variant
rs139409755	C	T	−0.16669	0.034691	1.55E-06	1032.08	C	T	0.008787	0.005135	0.087	AXIN1: Intron Variant
rs147734876	G	T	−0.31541	0.067812	3.30E-06	1074.83	G	T	−0.00061	0.01005	0.95	Not Reported in ClinVar
rs147793338	G	A	0.193076	0.042219	4.80E-06	1004	G	A	−0.00877	0.006263	0.16	Not Reported in ClinVar
rs150753765	A	G	−0.27205	0.055815	1.09E-06	1032.78	A	G	−0.00738	0.008281	0.37	Not Reported in ClinVar
rs2226602	A	T	0.075584	0.016183	3.00E-06	986.307	A	T	−0.00268	0.002348	0.25	RAB38: Intron Variant
rs3851566	A	G	0.062919	0.013736	4.63E-06	924.688	A	G	7.30E-05	0.002026	0.97	Not Reported in ClinVar
rs4841254	G	A	−0.06844	0.014352	1.85E-06	964.049	G	A	−0.0008	0.002103	0.7	GRIK4: Intron Variant
rs7103228	G	T	−1.40422	0.288972	1.18E-06	1913.97	G	A	−0.00043	0.002761	0.88	Delete
rs7103228	G	T	−1.40422	0.288972	1.18E-06	1913.97	G	T	0.012077	0.024698	0.62	GRIK4: Intron Variant
rs72820148	A	T	0.122046	0.026492	4.09E-06	946.795	A	T	0.002771	0.003931	0.48	Not Reported in ClinVar
rs73300965	C	A	−0.28484	0.060071	2.12E-06	1322.09	C	A	0.005937	0.00821	0.47	Not Reported in ClinVar
rs80054869	T	C	−0.30026	0.063508	2.27E-06	1030.74	T	C	−0.00157	0.009431	0.87	Not Reported in ClinVar
