# Supplementary material for: Causal pathway from telomere length to occurrence and 28-day mortality of sepsis: an observational and mendelian randomization study
Source: Aging (Albany NY). 2023 Aug 4;15(15):7727–40. doi: 10.18632/aging.204937 (PMC10457059; doi:10.18632/aging.204937)
Supplement: Supplementary Table 3 [file aging-15-204937-s002.docx]

| **Supplementary Table 3. Genetic Instrumental Variables of Reverse analysis and One-way analysis.** | | | | | | | | | | | | | |
| --- | --- | --- | --- | --- | --- | --- | --- | --- | --- | --- | --- | --- | --- |
| SNP | Effect  allele | Other  allele | LTL_exposure | | | | Sepsis_outcome | | | Sepsis(28day_death)_outcome | | | Note |
|  |  |  | beta | se | p_value | F | beta | se | p_value | beta | se | p_value |  |
| rs10024820 | T | C | 0.014444 | 0.002056 | 2.12E-12 | 37.53162 | 0.000446 | 0.013992 | 0.974579 | 0.034625 | 0.034167 | 0.310872 | BBS7 : Intron Variant |
| rs10112752 | G | A | 0.028752 | 0.002025 | 9.51E-46 | 169.1729 | 0.010131 | 0.013796 | 0.462712 | 0.028821 | 0.03371 | 0.392568 | TERF1 : 3 Prime UTR Variant |
| rs10150625 | C | T | 0.021552 | 0.003792 | 1.32E-08 | 26.51052 | 0.050843 | 0.026423 | 0.054333 | -0.02925 | 0.064503 | 0.650242 | Not Reported in ClinVar |
| rs10768147 | C | T | 0.011859 | 0.002007 | 3.43E-09 | 23.49036 | 0.012171 | 0.013683 | 0.373717 | 0.0276 | 0.03338 | 0.408318 | OR51E1 : Intron Variant |
| rs10773176 | A | G | 0.017201 | 0.002285 | 5.21E-14 | 42.82908 | 0.015488 | 0.015655 | 0.322494 | 0.035835 | 0.038222 | 0.348476 | Not Reported in ClinVar |
| rs10840270 | C | G | -0.01438 | 0.002125 | 1.30E-11 | 48.41943 | -0.00415 | 0.014554 | 0.775672 | -0.03244 | 0.035457 | 0.360209 | Not Reported in ClinVar |
| rs10845387 | G | A | 0.014121 | 0.002094 | 1.54E-11 | 27.90808 | -0.01594 | 0.014349 | 0.266626 | -0.02151 | 0.035001 | 0.538856 | Not Reported in ClinVar |
| rs10905255 | G | T | 0.018249 | 0.002031 | 2.58E-19 | 45.69432 | -0.00204 | 0.013883 | 0.883219 | -0.05482 | 0.033906 | 0.105939 | Not Reported in ClinVar |
| rs11085072 | C | T | 0.013181 | 0.002367 | 2.57E-08 | 39.20603 | -0.01009 | 0.016012 | 0.528728 | -0.01038 | 0.039062 | 0.790356 | SH3GL1 : Intron Variant |
| rs111527438 | T | C | -0.0125 | 0.00211 | 3.15E-09 | 18.90083 | 0.009081 | 0.014233 | 0.523458 | 0.072629 | 0.034761 | 0.036672 | ADAP2 : Intron Variant |
| rs111716290 | T | G | -0.03981 | 0.006638 | 2.00E-09 | 368.0232 | -0.02859 | 0.044763 | 0.523006 | -0.09343 | 0.109709 | 0.394406 | CFAP43 : Intron Variant |
| rs11190184 | G | C | 0.017217 | 0.002228 | 1.09E-14 | 68.89829 | -0.0158 | 0.015157 | 0.297173 | -0.00093 | 0.036986 | 0.97996 | Not Reported in ClinVar |
| rs112394943 | T | C | 0.019896 | 0.002816 | 1.61E-12 | 302.244 | 5.37E-05 | 0.019427 | 0.997796 | 0.028236 | 0.047502 | 0.552226 | Not Reported in ClinVar |
| rs112401627 | G | A | -0.05148 | 0.006722 | 1.87E-14 | 138.5381 | 0.024836 | 0.045063 | 0.581531 | 0.083669 | 0.109486 | 0.444747 | Not Reported in ClinVar |
| rs112511042 | T | C | 0.035143 | 0.004164 | 3.18E-17 | 53.92981 | 0.036236 | 0.028502 | 0.203605 | -0.01553 | 0.06926 | 0.822608 | SLX4 : Synonymous Variant |
| rs113800756 | A | C | 0.016558 | 0.002924 | 1.50E-08 | 15.60186 | 0.025397 | 0.02001 | 0.204366 | 0.040982 | 0.048792 | 0.400939 | Not Reported in ClinVar |
| rs11557154 | C | T | 0.034372 | 0.002985 | 1.13E-30 | 2846.965 | 0.004721 | 0.020506 | 0.81793 | -0.00884 | 0.049971 | 0.859574 | DCAF12 : Missense Variant |
| rs115610405 | C | A | 0.10951 | 0.00733 | 1.82E-50 | 56.9617 | 0.000636 | 0.049034 | 0.989659 | 0.072947 | 0.119788 | 0.542548 | RTEL1 : Missense Variant;RTEL1-TNFRSF6B : Noncoding Transcript Variant |
| rs115787626 | G | A | -0.05774 | 0.008455 | 8.59E-12 | 146.3064 | 0.05858 | 0.05692 | 0.303398 | 0.02728 | 0.139569 | 0.845033 | SMARCC1 : Intron Variant |
| rs11579626 | A | C | -0.02651 | 0.003578 | 1.26E-13 | 53.47837 | 0.011244 | 0.024578 | 0.647323 | -0.08284 | 0.059957 | 0.167078 | CHD1L : Intron Variant |
| rs11646283 | T | C | -0.01534 | 0.002039 | 5.30E-14 | 926.4431 | 0.009052 | 0.013941 | 0.516161 | -0.04988 | 0.034031 | 0.142681 | Not Reported in ClinVar |
| rs116539972 | C | T | 0.070457 | 0.008731 | 7.05E-16 | 786.6233 | -0.01162 | 0.058935 | 0.843672 | -0.19212 | 0.144538 | 0.183773 | Not Reported in ClinVar |
| rs117238689 | C | T | 0.058178 | 0.005851 | 2.71E-23 | 84.63089 | 0.012514 | 0.039366 | 0.750568 | -0.02824 | 0.095134 | 0.766551 | RTEL1 : Intron Variant;RTEL1-TNFRSF6B : Intron Variant |
| rs11736512 | A | G | -0.01895 | 0.003401 | 2.50E-08 | 41.31941 | 0.019832 | 0.022799 | 0.384382 | 0.04545 | 0.055584 | 0.41354 | NAF1 : Intron Variant |
| rs117407747 | C | T | -0.04505 | 0.006117 | 1.77E-13 | 377.2434 | 0.029274 | 0.041104 | 0.476344 | -0.06887 | 0.100052 | 0.491254 | Not Reported in ClinVar |
| rs11745132 | G | T | -0.0709 | 0.011937 | 2.85E-09 | 13.68551 | 0.140263 | 0.07984 | 0.07895 | 0.351086 | 0.19471 | 0.071369 | ZDHHC11 : Intron Variant |
| rs11746381 | T | C | -0.033 | 0.00431 | 1.93E-14 | 141.8411 | 0.006872 | 0.029087 | 0.813223 | 0.00186 | 0.070841 | 0.979057 | Not Reported in ClinVar |
| rs117469989 | T | G | 0.027909 | 0.004993 | 2.28E-08 | 64.33167 | 0.01222 | 0.033409 | 0.714536 | -0.08194 | 0.081661 | 0.315677 | Not Reported in ClinVar |
| rs117630647 | G | A | -0.05957 | 0.007204 | 1.36E-16 | 10.35057 | 0.048839 | 0.048054 | 0.30947 | 0.09093 | 0.11753 | 0.439123 | POT1-AS1 : Intron Variant |
| rs11769630 | T | A | 0.025681 | 0.003895 | 4.29E-11 | 90.69843 | 0.013878 | 0.026233 | 0.596778 | 0.016577 | 0.064088 | 0.7959 | Not Reported in ClinVar |
| rs117955747 | C | T | 0.073193 | 0.00747 | 1.15E-22 | 20.54245 | 0.007606 | 0.051905 | 0.883492 | -0.03145 | 0.125893 | 0.802754 | Not Reported in ClinVar |
| rs12369950 | T | C | 0.017831 | 0.002902 | 8.04E-10 | 45.1391 | 0.022599 | 0.019889 | 0.255847 | -0.06124 | 0.04844 | 0.206108 | Not Reported in ClinVar |
| rs12412214 | G | A | 0.024517 | 0.002227 | 3.42E-28 | 133.0267 | 0.020648 | 0.01515 | 0.172891 | 0.015471 | 0.036989 | 0.675751 | Not Reported in ClinVar |
| rs12572897 | G | A | 0.032149 | 0.002978 | 3.56E-27 | 472.8546 | 0.041407 | 0.020702 | 0.045481 | 0.076331 | 0.050483 | 0.130526 | NOC3L : Missense Variant |
| rs12615793 | G | A | -0.04516 | 0.002879 | 1.84E-55 | 1413.717 | 0.002256 | 0.019632 | 0.908529 | 0.032388 | 0.04788 | 0.498762 | ACYP2 : Intron Variant |
| rs12638862 | A | G | 0.086123 | 0.002279 | 0 | 546.5979 | -0.00423 | 0.015575 | 0.785995 | 0.018654 | 0.038064 | 0.62409 | Not Reported in ClinVar |
| rs1291143 | A | C | -0.04931 | 0.002799 | 1.79E-69 | 260.3285 | 0.037933 | 0.019362 | 0.0501 | 0.043432 | 0.047382 | 0.359335 | SAMHD1 : Intron Variant |
| rs13037426 | C | A | -0.03914 | 0.004665 | 4.80E-17 | 15.86712 | -0.0253 | 0.031306 | 0.419011 | -0.0518 | 0.075997 | 0.495525 | Not Reported in ClinVar |
| rs13062095 | T | C | -0.01386 | 0.002141 | 9.74E-11 | 94.21336 | 0.001742 | 0.014513 | 0.904437 | -0.01287 | 0.035431 | 0.716511 | Not Reported in ClinVar |
| rs13129697 | T | G | -0.01723 | 0.002241 | 1.49E-14 | 11.88827 | 0.00877 | 0.015421 | 0.569549 | 0.020068 | 0.037692 | 0.594433 | SLC2A9 : Intron Variant |
| rs13230646 | T | C | 0.017328 | 0.002324 | 8.87E-14 | 12.31587 | 0.003982 | 0.015808 | 0.801111 | 0.018167 | 0.038621 | 0.638086 | Not Reported in ClinVar |
| rs1332941 | A | G | -0.02566 | 0.002732 | 5.88E-21 | 40.42386 | 0.004108 | 0.018753 | 0.82659 | -0.00483 | 0.045795 | 0.916079 | Not Reported in ClinVar |
| rs137901416 | G | A | -0.04572 | 0.003324 | 4.66E-43 | 23.84848 | -0.02312 | 0.022484 | 0.303841 | -0.12456 | 0.054931 | 0.023355 | DCAF4 : Intron Variant |
| rs138017839 | T | C | 0.054115 | 0.007077 | 2.07E-14 | 276.8459 | -0.05099 | 0.047999 | 0.288071 | -0.04086 | 0.11663 | 0.726113 | Not Reported in ClinVar |
| rs138895564 | C | T | -0.18029 | 0.011066 | 1.13E-59 | 780.5907 | 0.057199 | 0.074587 | 0.443159 | 0.1577 | 0.183143 | 0.389198 | TERT : Intron Variant |
| rs139669835 | C | T | 0.061256 | 0.010535 | 6.07E-09 | 34.83234 | 0.017621 | 0.074106 | 0.812047 | -0.09429 | 0.183317 | 0.606988 | YES1 : Intron Variant |
| rs139795227 | A | C | -0.05994 | 0.008732 | 6.71E-12 | 168.7201 | -0.0114 | 0.05832 | 0.844957 | -0.18969 | 0.141844 | 0.181124 | GLMN : Intron Variant;RPAP2 : Intron Variant |
| rs144204502 | C | T | 0.100574 | 0.009134 | 3.37E-28 | 22.35198 | -0.00314 | 0.062012 | 0.959582 | -0.17709 | 0.151571 | 0.242655 | AFMID : Intron Variant;TK1 : 5 Prime UTR Variant |
| rs145011414 | G | A | -0.02903 | 0.005272 | 3.68E-08 | 470.0462 | 0.060224 | 0.035398 | 0.088881 | 0.082959 | 0.086337 | 0.336613 | SAMHD1 : Intron Variant |
| rs146546514 | C | A | -0.08035 | 0.008261 | 2.32E-22 | 16.61568 | 0.032865 | 0.055963 | 0.557033 | -0.04645 | 0.136553 | 0.733748 | LRRIQ4 : Intron Variant |
| rs148297846 | G | A | -0.03575 | 0.00363 | 6.80E-23 | 85.69074 | -0.02389 | 0.024868 | 0.336738 | 0.06115 | 0.060626 | 0.313147 | Not Reported in ClinVar |
| rs16978074 | T | G | -0.01916 | 0.002609 | 2.04E-13 | 21.42893 | -0.00962 | 0.017708 | 0.586824 | 0.039645 | 0.043217 | 0.358953 | LINC01478 : Intron Variant |
| rs17126584 | G | A | -0.01579 | 0.002725 | 6.90E-09 | 50.52236 | -0.00971 | 0.01882 | 0.605949 | -0.15206 | 0.045971 | 0.000941 | NUMB : Intron Variant |
| rs17185038 | C | G | 0.025779 | 0.004058 | 2.12E-10 | 12.37688 | -0.01583 | 0.027308 | 0.562167 | 0.069197 | 0.066861 | 0.300702 | RPA2 : Intron Variant |
| rs17445108 | G | A | 0.016892 | 0.00301 | 2.00E-08 | 80.45382 | 0.022337 | 0.020214 | 0.269149 | 0.027686 | 0.049276 | 0.574213 | PTGES3 : Non-Coding Transcript Variant |
| rs17677991 | C | G | -0.02227 | 0.002108 | 4.45E-26 | 75.178 | -0.00118 | 0.014388 | 0.93439 | 0.03426 | 0.035133 | 0.329486 | MGA : Missense Variant |
| rs17803849 | C | T | -0.02732 | 0.002035 | 4.24E-41 | 43.56629 | -0.00219 | 0.013938 | 0.875223 | -0.01656 | 0.033977 | 0.625962 | UNC80 : Intron Variant |
| rs17824845 | C | T | 0.013653 | 0.00201 | 1.10E-11 | 231.1958 | 0.012893 | 0.013712 | 0.347082 | 0.003747 | 0.033456 | 0.910829 | PPP1R36 : Intron Variant |
| rs17825740 | G | C | -0.03517 | 0.005276 | 2.61E-11 | 47.84456 | -0.01505 | 0.035521 | 0.671785 | -0.04596 | 0.087009 | 0.597308 | ZGPAT : Intron Variant |
| rs17843641 | A | G | -0.01483 | 0.002558 | 6.72E-09 | 237.5356 | -0.03585 | 0.017522 | 0.040728 | -0.0797 | 0.042782 | 0.062454 | HLA-DQA1 : Intron Variant |
| rs181412030 | A | T | 0.062198 | 0.009996 | 4.90E-10 | 189.566 | -0.06383 | 0.066865 | 0.33979 | -0.06025 | 0.16357 | 0.712613 | PHC3 : Intron Variant |
| rs182059586 | T | C | 0.057116 | 0.006809 | 4.91E-17 | 941.1126 | -0.04969 | 0.045698 | 0.276859 | -0.14216 | 0.111136 | 0.200848 | PARN : Intron Variant |
| rs183553155 | G | A | -0.07078 | 0.010153 | 3.12E-12 | 12.77672 | -0.00169 | 0.067773 | 0.980077 | 0.001189 | 0.166108 | 0.99429 | GLG1 : Intron Variant |
| rs183706774 | G | A | 0.021981 | 0.003583 | 8.52E-10 | 36.9984 | 0.020566 | 0.023995 | 0.391399 | 0.104507 | 0.058645 | 0.074745 | PSEN1 : Intron Variant |
| rs185174247 | G | A | -0.03728 | 0.004351 | 1.06E-17 | 704.315 | -0.0274 | 0.030152 | 0.36347 | 0.030862 | 0.073854 | 0.676035 | Not Reported in ClinVar |
| rs190110587 | C | T | 0.063111 | 0.011493 | 3.98E-08 | 14.28553 | 0.083825 | 0.076717 | 0.274544 | -0.30365 | 0.187243 | 0.104876 | TERT : Intron Variant |
| rs1907702 | G | A | -0.01502 | 0.002427 | 5.94E-10 | 69.72579 | -0.00911 | 0.016575 | 0.582599 | 0.047236 | 0.040553 | 0.244105 | KITLG : Intron Variant |
| rs1957937 | A | T | -0.02094 | 0.002734 | 1.88E-14 | 188.8889 | -0.02096 | 0.018869 | 0.266597 | -0.0245 | 0.046019 | 0.594432 | TCL1A : 2KB Upstream Variant LOC107984703 : 2KB Upstream Variant |
| rs1985369 | A | G | 0.031189 | 0.00301 | 3.63E-25 | 24.71177 | -0.04351 | 0.020605 | 0.034742 | -0.07434 | 0.050291 | 0.139339 | Not Reported in ClinVar |
| rs201125976 | G | A | 0.035392 | 0.004972 | 1.10E-12 | 121.4686 | 0.090751 | 0.033498 | 0.006746 | 0.167611 | 0.08142 | 0.039532 | HLA-DRB1 : Synonymous Variant |
| rs2056726 | G | A | 0.022808 | 0.002436 | 7.87E-21 | 48.39764 | 0.020968 | 0.016503 | 0.203886 | 0.015088 | 0.040254 | 0.707796 | STAG3 : Intron Variant |
| rs2069536 | A | G | 0.014756 | 0.002314 | 1.79E-10 | 65.75722 | -0.02916 | 0.015681 | 0.062946 | 0.004743 | 0.038326 | 0.90151 | CDK3 : Intron Variant TEN1-CDK3 : Intron Variant |
| rs2183509 | T | C | -0.04128 | 0.005405 | 2.23E-14 | 6.322394 | -0.00395 | 0.039021 | 0.919349 | -0.04584 | 0.095204 | 0.630165 | CFAP58 : Intron Variant |
| rs2230590 | T | C | 0.015802 | 0.002008 | 3.56E-15 | 58.94714 | 0.011269 | 0.01368 | 0.41008 | -0.03291 | 0.03343 | 0.324902 | MST1R : Missense Variant LOC102724438 : 2KB Upstream Variant |
| rs2276182 | C | G | -0.02335 | 0.002042 | 2.84E-30 | 117.0556 | 0.026976 | 0.01394 | 0.052966 | -0.00463 | 0.033983 | 0.89168 | POLI : Intron Variant |
| rs2282764 | A | G | 0.022423 | 0.002894 | 9.30E-15 | 19.36037 | -0.04746 | 0.02016 | 0.018575 | -0.08036 | 0.049306 | 0.103147 | MXD4 : Intron Variant |
| rs2293579 | G | A | 0.012915 | 0.002055 | 3.27E-10 | 46.84371 | 0.002735 | 0.014013 | 0.84524 | -0.04599 | 0.034178 | 0.178452 | PSMC3 : Intron Variant |
| rs2306646 | G | C | 0.020942 | 0.002019 | 3.31E-25 | 45.19651 | -0.00464 | 0.013784 | 0.73649 | -0.00245 | 0.03363 | 0.942 | being palindromic |
| rs2555104 | A | C | 0.013972 | 0.002035 | 6.61E-12 | 131.9675 | -0.02361 | 0.013804 | 0.087191 | -0.04451 | 0.033658 | 0.185983 | Not Reported in ClinVar |
| rs2763979 | C | T | 0.027771 | 0.002081 | 1.26E-40 | 109.6693 | 0.018186 | 0.014252 | 0.201951 | 0.030531 | 0.03481 | 0.380437 | HSPA1B : 2KB Upstream Variant |
| rs28502153 | C | A | 0.021592 | 0.002062 | 1.18E-25 | 29.93542 | -0.02454 | 0.014077 | 0.081287 | -0.04859 | 0.034286 | 0.156382 | GAB4 : Stop Gained |
| rs28711261 | A | G | -0.01674 | 0.002963 | 1.60E-08 | 38.59209 | -0.01362 | 0.020374 | 0.503966 | -0.01301 | 0.049665 | 0.793416 | CTCF : Intron Variant |
| rs2977608 | A | C | -0.01295 | 0.002337 | 3.02E-08 | 89.72709 | -0.03586 | 0.016087 | 0.025795 | -0.04461 | 0.039275 | 0.25599 | LINC01128 : Intron Variant |
| rs3093888 | G | A | 0.028973 | 0.004525 | 1.52E-10 | 36.22591 | 0.03329 | 0.031188 | 0.285785 | -0.0018 | 0.075865 | 0.981083 | PARP2 : Intron Variant；RPPH1 : 2KB Upstream Variant |
| rs310618 | T | C | 0.013797 | 0.002156 | 1.55E-10 | 88.17788 | 0.015346 | 0.014747 | 0.298039 | 0.019376 | 0.03597 | 0.590115 | EEF1A2 : Intron Variant |
| rs3129162 | T | G | 0.021421 | 0.00241 | 6.24E-19 | 54.71867 | -0.00933 | 0.016423 | 0.570109 | -0.01936 | 0.040099 | 0.62927 | Not Reported in ClinVar |
| rs34003787 | C | T | 0.024052 | 0.003584 | 1.92E-11 | 18.14412 | -0.07385 | 0.024462 | 0.002535 | -0.10592 | 0.059748 | 0.076257 | ZFHX3 : Intron Variant |
| rs34187696 | C | A | 0.012685 | 0.002227 | 1.23E-08 | 42.25105 | 0.012984 | 0.015073 | 0.388999 | 0.010029 | 0.036769 | 0.785049 | ZSCAN5A : Missense Variant |
| rs34842491 | T | C | 0.014336 | 0.002243 | 1.64E-10 | 991.535 | -0.00165 | 0.015348 | 0.914552 | 0.011921 | 0.037512 | 0.750653 | TYMSOS : Intron Variant |
| rs35640778 | G | A | 0.209011 | 0.007021 | 9.57E-195 | 52.56588 | -0.0769 | 0.047236 | 0.103518 | 0.008705 | 0.115323 | 0.939828 | RTEL1 : Missense Variant;RTEL1-TNFRSF6B : Noncoding Transcript Variant |
| rs35661976 | C | T | -0.03802 | 0.006971 | 4.92E-08 | 22.94713 | 0.061841 | 0.04797 | 0.197342 | -0.0946 | 0.116695 | 0.417576 | SLC6A18 : Synonymous Variant |
| rs35671754 | G | T | -0.01247 | 0.002199 | 1.40E-08 | 897.2912 | -0.0078 | 0.014969 | 0.602342 | -0.06008 | 0.036545 | 0.100182 | ATIC : Intron Variant |
| rs35902944 | G | C | -0.0634 | 0.00241 | 1.49E-152 | 85.83249 | 0.001589 | 0.016371 | 0.922659 | 0.005163 | 0.040024 | 0.897353 | RTEL1 : Intron Variant; RTEL1-TNFRSF6B : Intron Variant |
| rs3757387 | T | C | 0.020273 | 0.002015 | 8.21E-24 | 57.15538 | -0.01404 | 0.013729 | 0.3063 | -0.04764 | 0.033519 | 0.155263 | IRF5 : 2KB Upstream Variant |
| rs3768321 | G | T | 0.015576 | 0.002557 | 1.12E-09 | 19.05827 | 0.033853 | 0.017209 | 0.049162 | 0.041214 | 0.041911 | 0.325434 | PABPC4 : Intron Variant；PABPC4-AS1 : Intron Variant |
| rs38664 | T | C | 0.012247 | 0.002064 | 2.98E-09 | 5.883429 | 0.014159 | 0.014004 | 0.312002 | -0.00046 | 0.034168 | 0.989285 | Not Reported in ClinVar |
| rs3891167 | A | G | 0.042569 | 0.002396 | 1.20E-70 | 50.31609 | -0.01734 | 0.016302 | 0.2874 | -0.02383 | 0.039751 | 0.54889 | TYMS : Intron Variant；TYMSOS : 2KB Upstream Variant |
| rs41269079 | T | A | -0.01536 | 0.00255 | 1.70E-09 | 67.39864 | 0.01939 | 0.017339 | 0.263458 | -0.00909 | 0.042247 | 0.829659 | BEST4 : Intron Variant |
| rs429358 | T | C | -0.01735 | 0.002771 | 3.82E-10 | 76.76438 | 0.022005 | 0.0189 | 0.24429 | 0.07063 | 0.046104 | 0.125528 | POE : Missense Variant |
| rs430939 | G | A | 0.018339 | 0.002585 | 1.31E-12 | 20.36995 | 4.76E-05 | 0.017435 | 0.997821 | 0.010526 | 0.042581 | 0.804749 | Not Reported in ClinVar |
| rs4362428 | C | A | 0.011628 | 0.002051 | 1.43E-08 | 402.3796 | -0.01416 | 0.013905 | 0.308513 | -0.01865 | 0.033949 | 0.582794 | SMG6 : Intron Variant |
| rs4416632 | G | A | 0.041289 | 0.004874 | 2.43E-17 | 25.8931 | 0.003513 | 0.032855 | 0.914852 | -0.0302 | 0.080432 | 0.707284 | SLC6A19 : Intron Variant |
| rs4498805 | G | T | -0.01506 | 0.002004 | 5.65E-14 | 16.67476 | 0.020875 | 0.013712 | 0.127898 | 0.027611 | 0.033508 | 0.409935 | SLC16A4 : Intron Variant |
| rs450962 | A | G | -0.01428 | 0.002454 | 5.89E-09 | 20.16303 | 0.027522 | 0.016563 | 0.096576 | -0.0252 | 0.040329 | 0.532015 | EIF3CL : Intron Variant |
| rs4530278 | G | T | -0.01388 | 0.002057 | 1.50E-11 | 47.17672 | 0.005137 | 0.014065 | 0.714939 | -0.02762 | 0.034354 | 0.421382 | Not Reported in ClinVar |
| rs4695407 | A | G | -0.01415 | 0.001999 | 1.46E-12 | 256.2685 | 0.004293 | 0.013675 | 0.75359 | -0.02167 | 0.033423 | 0.516732 | OCIAD1 : Intron Variant |
| rs4724 | G | A | 0.054745 | 0.003124 | 9.81E-69 | 39.68795 | -0.00353 | 0.021893 | 0.872039 | -0.08178 | 0.053451 | 0.126008 | NAA38 : Synonymous Variant；CYB5D1 : 2KB Upstream Variant |
| rs4743037 | C | T | -0.0148 | 0.002381 | 5.14E-10 | 144.0678 | 0.005601 | 0.016385 | 0.732462 | 0.016258 | 0.040103 | 0.685174 | ZNF462 : Intron Variant |
| rs4919611 | C | A | 0.025594 | 0.003156 | 5.12E-16 | 18.58308 | 0.02038 | 0.021497 | 0.343113 | 0.095715 | 0.052467 | 0.068108 | PPRC1 : Intron Variant |
| rs56178008 | T | A | -0.01437 | 0.002015 | 9.70E-13 | 10.49987 | -0.00591 | 0.013766 | 0.667521 | -0.02526 | 0.033606 | 0.45225 | being palindromic |
| rs56179563 | G | A | -0.01255 | 0.002093 | 2.02E-09 | 251.3961 | 0.006387 | 0.014162 | 0.652019 | 0.023324 | 0.034591 | 0.500144 | ZC3HC1 : Intron Variant |
| rs56194506 | C | T | -0.0327 | 0.003559 | 3.92E-20 | 706.1748 | 0.025644 | 0.024611 | 0.297421 | 0.02138 | 0.060341 | 0.723099 | Not Reported in ClinVar |
| rs563069803 | G | T | 0.057197 | 0.008635 | 3.50E-11 | 31.87206 | -0.02645 | 0.060707 | 0.663004 | 0.099601 | 0.148113 | 0.501289 | Not Reported in ClinVar |
| rs5742915 | T | C | -0.01934 | 0.002029 | 1.55E-21 | 32.83782 | 0.003738 | 0.01371 | 0.785145 | 0.007187 | 0.033457 | 0.829918 | PML : Missense Variant |
| rs5770797 | G | C | -0.01634 | 0.002782 | 4.22E-09 | 34.34887 | -0.0245 | 0.019274 | 0.203611 | -0.00877 | 0.046985 | 0.851891 | MAPK8IP2 : Intron Variant |
| rs6007020 | T | C | -0.01449 | 0.002096 | 4.77E-12 | 49.14038 | -0.00043 | 0.014285 | 0.976256 | -0.04629 | 0.034894 | 0.184663 | SMC1B : Intron Variant |
| rs6054257 | G | A | 0.014168 | 0.002477 | 1.07E-08 | 20.90928 | -0.00141 | 0.016965 | 0.933787 | -0.0131 | 0.041336 | 0.751303 | DEFB125 : 2KB Upstream Variant |
| rs6090466 | G | A | -0.02578 | 0.003419 | 4.63E-14 | 141.3233 | 0.020633 | 0.023443 | 0.378789 | -0.05125 | 0.057273 | 0.370839 | GMEB2 : Intron Variant |
| rs609953 | T | A | -0.01348 | 0.002068 | 7.02E-11 | 31.20133 | 0.012358 | 0.014082 | 0.380176 | -0.05696 | 0.03442 | 0.097954 | HYAL4 : Intron Variant |
| rs60998424 | T | G | 0.050491 | 0.006066 | 8.49E-17 | 602.6109 | 0.064952 | 0.042519 | 0.12661 | -0.0149 | 0.103809 | 0.885888 | Not Reported in ClinVar |
| rs611646 | T | A | 0.036831 | 0.002035 | 3.52E-73 | 308.9542 | 0.010253 | 0.013881 | 0.460139 | 0.009332 | 0.033955 | 0.783448 | ATM : Intron Variant |
| rs61736615 | G | A | 0.037189 | 0.005375 | 4.56E-12 | 75.97044 | 0.002396 | 0.036107 | 0.947095 | -0.01849 | 0.08789 | 0.833399 | RTEL1 : Missense Variant;RTEL1-TNFRSF6B : Noncoding Transcript Variant |
| rs61748181 | C | T | 0.059181 | 0.005954 | 2.79E-23 | 801.8513 | -0.03583 | 0.040167 | 0.372369 | 0.024892 | 0.098051 | 0.799601 | TERT : Missense Variant |
| rs61818036 | G | A | 0.018944 | 0.002696 | 2.11E-12 | 41.81059 | -0.01282 | 0.018664 | 0.492272 | -0.03484 | 0.045523 | 0.444141 | Not Reported in ClinVar |
| rs62046862 | C | A | -0.02419 | 0.002088 | 4.88E-31 | 13.52803 | 0.003032 | 0.014101 | 0.829763 | -0.00809 | 0.034384 | 0.813887 | BANP : Intron Variant |
| rs62053340 | C | T | 0.021305 | 0.002079 | 1.24E-24 | 87.13499 | 0.010021 | 0.014069 | 0.476286 | 0.035587 | 0.034315 | 0.2997 | CLEC18A : Intron Variant |
| rs6590343 | A | G | -0.01217 | 0.002014 | 1.51E-09 | 16.09279 | 0.008756 | 0.013663 | 0.521632 | 0.036399 | 0.033389 | 0.275646 | Not Reported in ClinVar |
| rs6659669 | C | T | 0.011709 | 0.002052 | 1.15E-08 | 26.62376 | -0.00115 | 0.01393 | 0.934205 | 0.034693 | 0.034013 | 0.307739 | Not Reported in ClinVar |
| rs6669563 | G | A | -0.01824 | 0.002025 | 2.13E-19 | 74.21918 | -0.02759 | 0.01378 | 0.045278 | -0.02691 | 0.033649 | 0.42381 | SPOCD1 : Missense Variant |
| rs66731853 | G | A | 0.017779 | 0.002154 | 1.54E-16 | 23.88583 | -0.01038 | 0.014587 | 0.476788 | 0.045844 | 0.035651 | 0.19847 | CDA : Intron Variant |
| rs6751209 | T | C | 0.014047 | 0.002485 | 1.57E-08 | 27.20885 | 0.006888 | 0.017043 | 0.686098 | 0.081737 | 0.041531 | 0.049059 | THADA : Intron Variant |
| rs6776756 | G | A | 0.017444 | 0.002037 | 1.11E-17 | 35.00855 | 0.015932 | 0.013916 | 0.252249 | 0.005882 | 0.033914 | 0.862305 | GATA2-AS1 : Intron Variant |
| rs6873104 | A | T | 0.024533 | 0.003354 | 2.59E-13 | 98.46627 | 0.025826 | 0.023463 | 0.271037 | 0.018102 | 0.057445 | 0.752676 | TENT2 : Intron Variant |
| rs7012816 | G | A | -0.01759 | 0.002965 | 3.03E-09 | 54.79271 | 0.028333 | 0.020389 | 0.164638 | 0.01867 | 0.049794 | 0.707693 | PRDM14 : Intron Variant |
| rs7209057 | G | A | -0.01182 | 0.002029 | 5.68E-09 | 31.65806 | 0.004955 | 0.013868 | 0.720837 | 0.036617 | 0.033823 | 0.278988 | LOC101928045 : Intron Variant |
| rs7218033 | C | T | 0.022657 | 0.002309 | 1.00E-22 | 57.3745 | -0.02159 | 0.015802 | 0.171866 | -0.03811 | 0.03859 | 0.323423 | SMYD4 : Intron Variant |
| rs72801474 | G | A | 0.020745 | 0.003524 | 3.93E-09 | 41.86482 | -0.01818 | 0.023605 | 0.441314 | -0.04137 | 0.057458 | 0.471558 | Not Reported in ClinVar |
| rs73154592 | A | T | 0.017462 | 0.002004 | 2.90E-18 | 53.70876 | 0.012301 | 0.013719 | 0.369906 | 0.058734 | 0.03344 | 0.079015 | being palindromic |
| rs73581419 | C | T | -0.02298 | 0.003242 | 1.34E-12 | 113.7032 | 0.011458 | 0.021911 | 0.601005 | -0.0488 | 0.053578 | 0.362373 | RAB2B : Intron Variant |
| rs7555872 | A | G | -0.02727 | 0.004987 | 4.57E-08 | 97.95453 | 0.079999 | 0.035241 | 0.023204 | 0.204774 | 0.086087 | 0.017374 | Not Reported in ClinVar |
| rs75664430 | C | G | 0.023518 | 0.002319 | 3.55E-24 | 128.6659 | 0.025303 | 0.015731 | 0.107726 | -0.01478 | 0.038407 | 0.700424 | VAMP2 : Intron Variant |
| rs75683534 | C | A | -0.04473 | 0.007619 | 4.36E-09 | 354.2696 | 0.005525 | 0.050773 | 0.913341 | 0.08573 | 0.124157 | 0.48988 | PIF1 : Stop Gained |
| rs76219171 | G | A | -0.03598 | 0.004317 | 7.78E-17 | 300.8294 | -0.02612 | 0.028951 | 0.366971 | 0.00234 | 0.070856 | 0.973652 | TENT4B : Intron Variant |
| rs762679 | T | A | -0.03101 | 0.00285 | 1.44E-27 | 179.0038 | 0.018137 | 0.019339 | 0.348319 | 0.02098 | 0.04728 | 0.657239 | MCM4 : Missense Variant |
| rs762810 | C | A | 0.020297 | 0.0021 | 4.27E-22 | 73.61638 | 0.025719 | 0.014258 | 0.071267 | 0.00255 | 0.034805 | 0.941602 | MAX : Noncoding Transcript Variant |
| rs76374777 | G | C | -0.04875 | 0.007993 | 1.07E-09 | 185.8531 | -0.02127 | 0.054109 | 0.69421 | -0.0831 | 0.130983 | 0.525797 | PHACTR4 : Intron Variant |
| rs76666449 | T | C | -0.02951 | 0.003332 | 8.17E-19 | 19.57102 | -0.01997 | 0.022598 | 0.376935 | -0.02454 | 0.055027 | 0.655654 | SRSF9 : Intron Variant |
| rs76883411 | G | C | 0.040101 | 0.006498 | 6.79E-10 | 14.08883 | -0.0245 | 0.045449 | 0.589814 | 0.087649 | 0.11076 | 0.428746 | ENOSF1 : Intron Variant |
| rs7705526 | C | A | -0.0776 | 0.002161 | 2.43E-282 | 840.3385 | 0.03519 | 0.014749 | 0.01704 | -0.01542 | 0.035989 | 0.668245 | TERT : Intron Variant |
| rs77426195 | G | A | -0.02419 | 0.003623 | 2.46E-11 | 132.9667 | 0.048179 | 0.024415 | 0.04846 | -0.0044 | 0.059598 | 0.941108 | Not Reported in ClinVar |
| rs7790856 | C | T | 0.04372 | 0.002205 | 1.80E-87 | 416.9778 | 0.000838 | 0.015064 | 0.955642 | -0.06741 | 0.036805 | 0.067025 | Not Reported in ClinVar |
| rs78491606 | A | C | 0.075631 | 0.007412 | 1.90E-24 | 642.3898 | -0.00486 | 0.049654 | 0.922108 | -0.04592 | 0.120281 | 0.702631 | SHQ1 : Missense Variant |
| rs78701368 | A | G | 0.070438 | 0.007913 | 5.51E-19 | 848.5515 | -0.10635 | 0.053436 | 0.046574 | -0.19807 | 0.130258 | 0.128354 | ZBTB46 : 2KB Upstream Variant |
| rs78933533 | G | A | 0.03606 | 0.005308 | 1.10E-11 | 255.3609 | 0.066087 | 0.036585 | 0.070852 | 0.143994 | 0.089098 | 0.106064 | HLA-DQA1 : 2KB Upstream Variant |
| rs79127610 | G | A | -0.058 | 0.006789 | 1.30E-17 | 764.7636 | 0.019108 | 0.045682 | 0.675748 | -0.03451 | 0.111915 | 0.757782 | Not Reported in ClinVar |
| rs79280861 | G | C | -0.03737 | 0.00474 | 3.20E-15 | 171.8407 | 0.033619 | 0.032291 | 0.29781 | 0.071157 | 0.078763 | 0.366297 | Not Reported in ClinVar |
| rs79683019 | C | A | 0.071415 | 0.008896 | 9.91E-16 | 971.5764 | 0.030185 | 0.05992 | 0.614436 | -0.04239 | 0.146298 | 0.771989 | Not Reported in ClinVar |
| rs79755767 | G | A | -0.02787 | 0.003433 | 4.76E-16 | 179.9912 | 0.041743 | 0.023071 | 0.0704 | 0.076045 | 0.056209 | 0.176089 | Not Reported in ClinVar |
| rs8006485 | G | T | -0.01915 | 0.002002 | 1.16E-21 | 56.72043 | 0.009189 | 0.013704 | 0.502513 | -0.01717 | 0.033466 | 0.607957 | PPP4R3A : Intron Variant |
| rs80150989 | A | T | -0.02794 | 0.004638 | 1.70E-09 | 94.49981 | -0.00982 | 0.031286 | 0.753718 | -0.12416 | 0.07607 | 0.102654 | PRPF6 : Intron Variant |
| rs80324517 | G | A | -0.03965 | 0.004663 | 1.84E-17 | 54.46338 | 0.017524 | 0.031449 | 0.577379 | -0.15602 | 0.076502 | 0.041405 | LOC285766 : Intron Variant |
| rs8051249 | T | C | -0.01597 | 0.002851 | 2.11E-08 | 53.71817 | 0.003254 | 0.019756 | 0.869155 | -0.01825 | 0.048144 | 0.70467 | PARN : Intron Variant |
| rs8053839 | G | T | 0.013921 | 0.002046 | 1.03E-11 | 30.49595 | 0.018481 | 0.013865 | 0.18255 | -0.01962 | 0.033849 | 0.562191 | LONP2 : Intron Variant；SIAH1 : 3 Prime UTR Variant |
| rs8088824 | C | T | -0.02577 | 0.002357 | 8.14E-28 | 14.04559 | -0.00039 | 0.01616 | 0.980939 | -0.02704 | 0.039448 | 0.493067 | Not Reported in ClinVar |
| rs8102497 | G | A | 0.014965 | 0.002023 | 1.40E-13 | 34.19448 | -0.00877 | 0.013815 | 0.525755 | -0.03178 | 0.033656 | 0.34509 | Not Reported in ClinVar |
| rs8105767 | A | G | -0.03284 | 0.002201 | 2.49E-50 | 189.5308 | -0.00394 | 0.015122 | 0.794295 | -0.0275 | 0.036932 | 0.456495 | LOC112268248 : Intron Variant |
| rs871134 | C | T | 0.018299 | 0.002026 | 1.71E-19 | 11.17023 | -0.01163 | 0.013842 | 0.400726 | 0.035641 | 0.033733 | 0.290716 | CCDC96 : Stop Gained；TADA2B : 2KB Upstream Variant；LOC100129931 : Intron Variant |
| rs9271657 | T | C | 0.021592 | 0.002545 | 2.16E-17 | 8.147864 | 0.004986 | 0.017374 | 0.774128 | 0.021866 | 0.042337 | 0.605525 | Not Reported in ClinVar |
| rs932002 | C | T | 0.040205 | 0.002797 | 7.31E-47 | 54.719 | 0.016231 | 0.018985 | 0.392585 | 0.039505 | 0.046377 | 0.394312 | PARP1 : Intron Variant |
| rs939916 | G | A | -0.02418 | 0.002167 | 6.63E-29 | 25.23252 | 0.005561 | 0.014819 | 0.707468 | -0.07646 | 0.03616 | 0.034462 | Not Reported in ClinVar |
| rs9419958 | T | C | 0.08101 | 0.002938 | 2.64E-167 | 295.9252 | -0.02995 | 0.020216 | 0.138423 | -0.0521 | 0.049351 | 0.291098 | STN1 : Intron Variant |
| rs9600019 | C | T | -0.01271 | 0.002131 | 2.43E-09 | 35.88805 | 0.008457 | 0.014507 | 0.559939 | 0.050406 | 0.035378 | 0.154214 | BORA : Intron Variant |
| rs965109 | C | T | 0.101702 | 0.006482 | 1.77E-55 | 2282.238 | -0.00826 | 0.043738 | 0.850143 | 0.011297 | 0.10639 | 0.915435 | ACYP2 : Intron Variant |
| rs9878436 | C | T | 0.014341 | 0.002018 | 1.20E-12 | 31.95748 | -0.03818 | 0.013789 | 0.00562 | 0.019895 | 0.033619 | 0.554013 | CEP70 : Synonymous Variant |
| rs9923119 | T | C | -0.0177 | 0.002403 | 1.80E-13 | 39.9621 | 0.032831 | 0.016449 | 0.045934 | 0.00022 | 0.040185 | 0.995624 | Not Reported in ClinVar |
